# Association of low fasting C-peptide levels with cardiovascular risk, visit-to-visit glucose variation and severe hypoglycemia in the Veterans Affairs Diabetes Trial (VADT)

**DOI:** 10.1186/s12933-021-01418-z

**Published:** 2021-12-08

**Authors:** Juraj Koska, Daniel S. Nuyujukian, Gideon D. Bahn, Jin J. Zhou, Peter D. Reaven

**Affiliations:** 1 Phoenix VA Health Care System, 650 E. Indian School Road/CS111E, Phoenix, AZ 85012-1892 USA; 2 Department of Epidemiology and Biostatistics, University of Arizona, Tucson, AZ USA; 3 Hines Veterans Affairs Cooperative Studies Program Coordinating Center, Edward Hines, Jr. Veterans Affairs Hospital, Hines, IL USA; 4 University of California, Los Angeles, Los Angeles, CA USA; 5 University of Arizona-College of Medicine, Phoenix, AZ USA

## Abstract

**Aims:**

Low C-peptide levels, indicating beta-cell dysfunction, are associated with increased within-day glucose variation and hypoglycemia. In advanced type 2 diabetes, severe hypoglycemia and increased glucose variation predict cardiovascular (CVD) risk. The present study examined the association between C-peptide levels and CVD risk and whether it can be explained by visit-to-visit glucose variation and severe hypoglycemia.

**Materials and methods:**

Fasting C-peptide levels at baseline, composite CVD outcome, severe hypoglycemia, and visit-to-visit fasting glucose coefficient of variation (CV) and average real variability (ARV) were assessed in 1565 Veterans Affairs Diabetes Trial participants.

**Results:**

There was a U-shaped relationship between C-peptide and CVD risk with increased risk with declining levels in the low range (< 0.50 nmol/l, HR 1.30 [95%CI 1.05–1.60], p = 0.02) and with rising levels in the high range (> 1.23 nmol/l, 1.27 [1.00–1.63], p = 0.05). C-peptide levels were inversely associated with the risk of severe hypoglycemia (OR 0.68 [0.60–0.77]) and visit-to-visit glucose variation (CV, standardized beta-estimate − 0.12 [SE 0.01]; ARV, − 0.10 [0.01]) (p < 0.0001 all). The association of low C-peptide levels with CVD risk was independent of cardiometabolic risk factors (1.48 [1.17–1.87, p = 0.001) and remained associated with CVD when tested in the same model with severe hypoglycemia and glucose CV.

**Conclusions:**

Low C-peptide levels were associated with increased CVD risk in advanced type 2 diabetes. The association was independent of increases in glucose variation or severe hypoglycemia. C-peptide levels may predict future glucose control patterns and CVD risk, and identify phenotypes influencing clinical decision making in advanced type 2 diabetes.

**Supplementary Information:**

The online version contains supplementary material available at 10.1186/s12933-021-01418-z.

## Introduction

The natural history of type 2 diabetes is characterized by distinct patterns of beta-cell function. In the early phase of the disease, beta-cell function is relatively well preserved and insulin secretion is increased as part of the compensatory requirement for higher insulin levels to overcome insulin resistance [1]. As diabetes progresses, beta-cell function gradually deteriorates, frequently requiring exogenous insulin supplementation to maintain glycemic target [2]. Because up to 80% of secreted insulin is cleared during its first passage through the liver, blood levels of C-peptide, a by-product of proinsulin cleavage, have been used as a better estimate of beta-cell function [3, 4].

Several studies previously examined the relationship between C-peptide levels and complications in type 2 diabetes with inconsistent results. In mixed populations of people with and without diabetes or in those with early diabetes, higher C-peptide levels were associated with worse metabolic risk profiles and higher risk of diabetes complications [5–8]. In contrast, persons with more chronic type 2 diabetes and low C-peptide levels have a greater prevalence of diabetes complications, despite more favorable non-glycemic risk factors [9]. In the Veterans Affairs Diabetes Trial (VADT), longer duration of type 2 diabetes was associated with lower C-peptide levels and with greater CVD risk during intensive glucose lowering treatment [10]. Why intensive lowering was particularly harmful in those with low C-peptide was not examined.

In type 2 diabetes, low C-peptide levels, indicating more severe beta-cell dysfunction, are associated with a broad spectrum of disturbances in glucose control, including higher levels of hemoglobin A_1c_ (HbA_1c_), greater within-day glucose variation, and increased incidence of hypoglycemia [9, 11–15]. Recently, our group and others have shown that greater visit-to-visit glucose variation in individuals with type 2 diabetes at high risk of cardiovascular disease (CVD) was associated with increased risk of CVD events [16, 17]. High hemoglobin glycation index, reflecting mismatch between predicted and observed HbA_1c_ which can result from glucose variation, has also been linked with CVD and mortality [18]. In post-hoc analyses of intensive glucose lowering trials, incidence of CVD events or death was also directly linked to severe hypoglycemia [19–21].

Thus, in this post-hoc analysis of the VADT cohort we examined whether low C-peptide levels were associated with increased visit-to-visit glucose variation or risk of severe hypoglycemia during glucose-lowering trial. We then tested whether these disturbances in glucose regulation contribute, in turn, to increased CVD risk in those with low C-peptide.

## Research design and methods

This study reflects a post-hoc analysis of data from the Veterans Affairs Diabetes Trial (VADT, NCT00032487). As part of the study baseline assessment, C-peptide levels were measured in 1693 of the 1791 eventual VADT participants at the DRTC Core Lab, University of Chicago, as described previously [22]. The design and principal results of the VADT have been described previously [23, 24]. Briefly, military veterans with type 2 diabetes and suboptimal glycemic control (HbA_1c_ > 58 mmol/l [7.5%]) were randomized to either intensive or standard glucose control. The two groups were treated with similar diabetes medications (but at different doses) with a goal of achieving an absolute difference of 7.1 mmol/mol (1.5%) HbA_1c_ between treatment groups. HbA_1c_ and fasting glucose were measured every 3 months up to a maximum of 84 months. In both groups, HbA_1c_ decreased after 3 months and stabilized after 6 months of the trial with a 7.1 mmol/mol median HbA_1c_ separation between the groups. To eliminate this rapid initial (per protocol) reduction in glucose levels and to ensure a reliable calculation of glycemic variation, only 1565 participants with at least two measurements of fasting glucose after the 6-month visit were included in the analyses.

The primary CVD outcome in the VADT, and this analysis, was time to the first occurrence of any one of a documented and independently adjudicated myocardial infarction, stroke, death from cardiovascular causes, new or worsening congestive heart failure, surgical intervention for cardiac, cerebrovascular, or peripheral vascular disease, inoperable coronary artery disease, and amputation for ischemic gangrene. As detailed previously, fasting glucose variation was calculated as the visit-to-visit coefficient of variation (CV) and average real variability (ARV) [16]. Severe hypoglycemia was defined as a self-reported episode of a low blood glucose value accompanied by confusion requiring assistance from another person or loss of consciousness [19]. The modified updated Charlson comorbidity index was calculated to reflect overall comorbidity level at baseline as indicated previously [25].

Statistical analyses were performed using SAS version 9.4 (SAS Inc., Cary, NC). A two-sided p < 0.05 was considered statistically significant. Non-normally distributed variables were natural log transformed to approximate normal distribution for the analyses. P-values of < 0.05 were considered statistically significant. To demonstrate the differences in baseline characteristics by C-peptide levels, the study population was categorized by quartiles of C-peptide values. Comparisons of variables in higher quartiles with those in the bottom quartile as reference were tested in each glucose-lowering group by one-way ANOVA for continuous variables, or by logistic regression for binary variables.

The association between baseline C-peptide levels and follow-up measures of glucose control was tested after adjusting for glucose-lowering group and then also adjusting for clinical and demographic factors, i.e., diabetes duration, age, race/ethnicity, HbA_1c_, body mass index (BMI) and estimated glomerular filtration rate (eGFR), and then also for insulin usage. Logistic regression was used to test the association between baseline C-peptide and any severe hypoglycemia occurrence. Linear mixed effect regression with random intercept was used to test the association between C-peptide levels and means of updated visit-to-visit glucose CV and ARV. Natural log-transformed values of baseline C-peptide levels and glucose variation measures were standardized to one SD to allow direct comparison of effects.

Cox proportional hazard analysis was used to test the association between baseline C-peptide levels and CVD risk. Non-linear relationship between continuous C-peptide levels and CVD risk was tested by the likelihood ratio test comparing the models with the linear term only with the model including both linear and cubic spline terms [26]. Restricted cubic splines using the percentile method with 3 knots was used to estimate the association between C-peptide levels and CVD. Threshold values for low and high C-peptide ranges were defined as values corresponding to hazard ratio of one. The models were first adjusted for glucose-lowering group and then also adjusted for CVD risk factors, including CVD history, diabetes duration, age, race/ethnicity, HbA_1c_, body mass index (BMI), estimated glomerular filtration rate (eGFR), and HDL and LDL cholesterol.

To test whether severe hypoglycemia and visit-to-visit glucose variation explain the association between low C-peptide levels and CVD, the effect of low C-peptide levels was assessed in combined models with visit-to-visit glucose variation and severe hypoglycemia calculated as time-dependent covariates until time of an event.

## Results

**Fig. 1:**
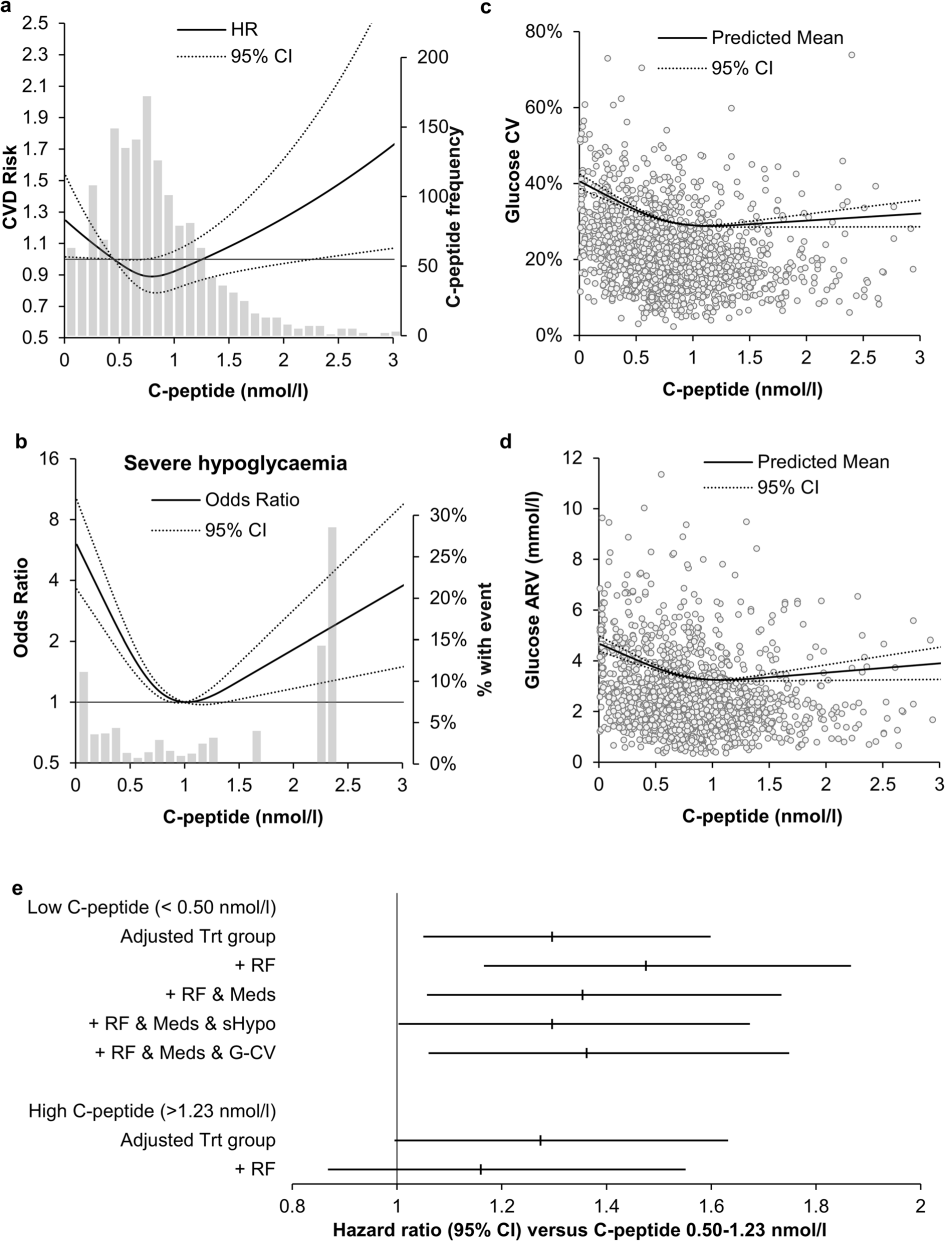
Association between baseline C-peptide levels and CVD risk, and follow-up measures of glucose control. **a** Restricted cubic splines curve and 95% CI of the relationship between C-peptide levels and CVD risk. Shaded region represents the frequency of C-peptide levels. **b** Restricted cubic splines curve and 95% CI of the relationship between C-peptide levels and severe hypoglycemia. Shaded region represents the percentage of hypoglycemic events. **c, d** Association between baseline C-peptide levels and updated mean visit-to-visit glucose CV and ARV. All models on panels A-D were adjusted for glucose-lowering group. **e** Hazard ratios (95% CI) for low (< 0.50 nmol/l) and high (> 1.23 nmol/l) C-peptide levels with CVD risk after adjustment for glucose lowering group (Trt group), clinical and demographic risk factors (RF), baseline insulin and sulphonylureas (SU) use (Meds), and glucose CV (G-CV) or severe hypoglycemia (sHypo). Low (< 0.50 nmol/l) and high C-peptide (> 1.23 nmol/l) ranges in analyses using restricted cubic splines were defined by C-peptide values corresponding to a hazard ratio of one (**a**)

At baseline, from the lowest to highest quartile of plasma C-peptide levels there was an increase in the prevalence of non-Hispanic Whites and a corresponding decrease in the percent self-reporting as African Americans, as well as increases in history of prior CVD, history of hypertension, body mass index (BMI), and plasma triglycerides (Table 1). We also observed decreases in diabetes duration, HbA_1c_, estimated glomerular filtration rate (eGFR) and HDL-cholesterol levels. In those with the lowest C-peptide levels (defined as the bottom quartile), there was a greater duration of diabetes, and slightly higher LDL cholesterol and HbA_1c_. However, many cardiovascular risk factors tended to be less prevalent (including a prior history of CVD, hypertension, low eGFR, elevated triglycerides and low HDL) or equally prevalent (e.g., smoking). Consistent with this, the UKPDS risk score was lowest in quartile 1 and highest in quartile 4. Importantly, the updated Charlson index as a measure of chronic comorbidity was not significantly different between the baseline C-peptide quartiles. Those with lower C-peptide levels were also more likely to receive insulin therapy and less likely to use metformin and sulphonylureas. Among those on insulin therapy, there was no association between total daily insulin dose and fasting C-peptide levels (Spearman correlation coefficient − 0.03, p = 0.4). In a multivariable regression model including characteristics that differed between the C-peptide quartiles, plasma C-peptide levels (as a continuous variable) were inversely associated with diabetes duration, use of insulin, plasma HDL cholesterol (all p < 0.0001) and eGFR (p = 0.003), and positively associated with plasma triglycerides, BMI, (both p < 0.0001), history of hypertension (p = 0.04) and use of sulphonylureas (p = 0.02) (data not shown). Over a median follow-up of 5.3 years, 459 participants developed a CVD event (median time to event 2.3 years; median time to censoring 5.7 years). There was a U-shaped relationship between continuous C-peptide levels and CVD risk (Fig. 1a). As C-peptide levels declined among those in the lower C-peptide range (< 0.50 nmol/l), risk for CVD increased. In contrast, as C-peptide levels increased within the higher C-peptide range (> 1.23 nmol/l) risk for CVD also increased (Fig. 1a). Similar relationship between C-peptide levels and CVD was observed in all 1,693 VADT individuals with baseline C-peptide measurement (n = 479 events) (Additional file 1). The risk of severe hypoglycemia and visit-to-visit glucose variation during VADT were also higher in those with lower C-peptide levels (all p < 0.0001, Fig. 1b–d). The inverse associations between C-peptide levels and both measures of glucose control remained significant after adjustment for clinical and demographic risk factors, and baseline insulin use (Table 2, all p < 0.0001). The association between lower C-peptide levels and CVD was significant after adjustment for clinical and demographic risk factors, baseline insulin and sulphonylureas use, and after adjustment for severe hypoglycemia or visit-to-visit glucose CV (Fig. 1e). In contrast, the association between higher C-peptide levels and CVD was attenuated after adjustment for clinical and demographic risk factors (Fig. 1e).

**Table 1:** Clinical and demographic characteristics at baseline of 1565 VADT participants included in present study by quartiles of baseline C-peptide levels

Quartile	Q1 (n = 385)	Q2 (n = 404)	Q3 (n = 389)	Q4 (n = 387)
Median [Range] (nmol/l)	0.26 [0.01–0.45]	0.60 [0.46–0.73]	0.87 [0.74–1.06]	1.36 [1.07–4.88]
Age (years)	60 ± 9	61 ± 8	60 ± 9	60 ± 8
Male sex (%)	97%	97%	97%	99%
Race/ethnicity				
Caucasian (%)	54%	55%	67%*	73%*
African American (%)	24%	21%	15%*	7%*
Hispanic (%)	17%	19%	15%	14%
Other (%)	5%	5%	3%	6%
Prior CVD (%)	35%	39%	44%*	45%*
History of Hypertension (%)	68%	73%	75%*	76%*
Body Mass Index (kg/m^2^)	30.1 ± 4.7	30.7 ± 4.3*	31.5 ± 4.3*	32.8 ± 4.3*
Systolic BP (mmHg)	131 ± 17	132 ± 16	134 ± 17*	130 ± 16
Diastolic BP (mmHg)	75 ± 10	76 ± 10	77 ± 10*	76 ± 10
Current smoker (%)	17%	15%	13%	19%*
Diabetes duration (years)	14 ± 8	12 ± 7*	11 ± 7*	9 ± 6*
Hemoglobin A_1c_ (%)	9.6 ± 1.6	9.4 ± 1.5*	9.4 ± 1.5	9.3 ± 1.3*
Hemoglobin A_1c_ (mmol/mol)	81 ± 18	79 ± 17*	79 ± 17	78 ± 14*
Fasting Glucose (mmol/l)	11.2 ± 4.2	11.4 ± 3.5*	11.6 ± 3.9*	11.1 ± 3.4
Insulin use (%)	78%	49%*	41%*	32%*
Metformin use (%)	73%	78%	82%*	88%*
Sulphonylureas use (%)	36%	41%	44%*	44%*
Rosiglitazone use (%)	89%	86%	83%*	84%
Total cholesterol (mmol/l)	4.7 ± 1.0	4.8 ± 1.6	4.8 ± 1.1	4.8 ± 1.2
LDL cholesterol (mmol/l)	2.9 ± 0.8	2.8 ± 0.8	2.8 ± 0.8*	2.7 ± 0.8*
HDL cholesterol (mmol/l)	1.05 ± 0.31	0.96 ± 0.26*	0.88 ± 0.22*	0.82 ± 20*
Triglycerides (mmol/l)	1.5 ± 0.7	1.8 ± 0.8*	2.1 ± 0.9*	2.3 ± 0.9*
Lipid-lowering drugs (%)	64%	68%	66%	70%
eGFR (ml/min/1.73m^2^)	83 ± 20	84 ± 21	82 ± 21	79 ± 24*
UKPDS risk score	0.020 ± 0.019	0.023 ± 0.023	0.027 ± 0.022*	0.032 ± 0.030*
Charlson Comorbidity Index	2.30 ± 0.27	2.32 ± 0.23	2.30 ± 0.28	2.28 ± 0.28

Data are means ± SD or percentages for quartiles (Q1, bottom quartile; Q2, 2nd quartile; Q3, 3rd quartile; Q4, top quartile). *p < 0.05 vs Q1 by linear (for natural log transformed continuous characteristics) or logistic (for categories) regression

**Table 2:** Relationship between baseline C-peptide levels and follow-up measurements of glucose control

	Model 1	Model 2	Model 3
Severe hypoglycemia^†^	0.68 (0.60, 0.77)*	0.70 (0.61, 0.80)*	0.77 (0.66, 0.88)*
Mean Glucose CV (1 SD)^‡^	− 0.12 (− 0.14, − 0.10)*	− 0.10 (− 0.12, − 0.08)*	− 0.07 (− 0.10, − 0.05)*
Mean Glucose ARV (1 SD)^‡^	− 0.12 (− 0.15, − 0.10)*	− 0.10 (− 0.13, − 0.08)*	− 0.08 (− 0.10, − 0.05)*

Outcomes were any occurrence of severe hypoglycemia, mean updated glucose coefficient of variation (CV) or average real variability (ARV). Model 1, adjusted for glucose-lowering group; Model 2, additionally adjusted for baseline age, race/ethnicity, diabetes duration, HbA_1c_, body mass index and eGFR; Model 3, additionally adjusted for insulin and sulphonylureas use. Data are Odds ratios (OR) or standardized β-estimates (95% CI) per 1 SD of C-peptide levels

^†^Logistic regression, ^‡^Mixed model linear regression with random intercept, *p < 0.05

## Discussion

In the present study of type 2 diabetes participants in the VADT, we found a significant association between both low and high baseline C-peptide levels and greater risk for CVD. The association between low C-peptide and CVD risk was present despite more favorable standard cardiovascular risk factors, such as BMI, history of hypertension, dyslipidemia, smoking, and prior CVD. As a result, adjustment for these typical risk factors further strengthened the association between low C-peptide levels and CVD. Low C-peptide levels were, however, associated with increased visit-to-visit glucose variation and severe hypoglycemia over 7.5 years of the VADT follow-up, regardless of the glycemic control targets in the glucose lowering trial. Each of these components of glucose control has been linked with CVD risk or mortality in glucose-lowering trials [16, 17, 19–21], and could explain in part the relationship of C-peptide with this outcome. Closer examination of these relationships revealed that low C-peptide levels were associated with CVD independently of both prospective glucose control measures, indicating other mechanisms may explain the association between low C-peptide levels and increased CVD risk.

Pathways by which low C-peptide concentrations may be related to CVD risk could include disturbances in glucose control that were not captured in the VADT, such as non-fasting glucose fluctuations or unrecognized hypoglycemic episodes. Alternatively, several previous experimental studies indicated direct beneficial effects of C-peptide on the vasculature, which could be lost in the setting of very low C-peptide levels. In animal models of type 1 diabetes, physiological supplementation of C-peptide protected against hyperglycemia-induced endothelial cell apoptosis [27], and normalized hyperglycemia-induced AMPKα dephosphorylation, ROS generation, and mitochondrial disorganization in aorta of diabetic mice [28]. Supraphysiological levels of C-peptide prevent smooth muscle cell proliferation and neointima formation [29], and in a small study of humans with type 1 diabetes, short-term C-peptide infusion improved myocardial blood flow and left-ventricular function [30].

To our knowledge, this is the first study to report an association between C-peptide levels and *long-term* visit-to-visit glucose variation. This is consistent with reports from several small cohorts of type 2 diabetes patients where within day variation was assessed by continuous glucose monitoring (CGM) [11–13]. The increase in visit-to-visit glucose variation in those in the bottom C-peptide quartile in our study remained significant after adjusting for baseline insulin use. Furthermore, there was no significant interaction of C-peptide quartiles with insulin use (versus no insulin use) in the association with glucose variation. Thus, these data indicate that the greater glucose variation in the low C-peptide group is likely not simply a function of insulin use, and rather reflects severe inability of beta cells to maintain glucose levels.

An additional interesting finding in this study was that even in this rather homogenous group of older individuals with advanced type 2 diabetes who remained hyperglycemic despite several diabetes medications, there was a strikingly different clinical phenotype across C-peptide levels. Those with the highest C-peptide values (defined as quartiles in the present analysis) had multiple characteristics of insulin resistance such as higher body weight, plasma triglycerides levels, prevalence of hypertension, and lower plasma HDL levels. Consistent with this risk profile, they also had a higher prevalence of CVD at study enrolment, and a positive relationship between C-peptide and CVD as one moved to higher C-peptide levels. These findings are in line with previous studies showing increased risk of microvascular and macrovascular complications, as well as all-cause mortality with higher concentrations of C-peptide in mixed populations (with and without type 2 diabetes) or in those with early type 2 diabetes, i.e., individuals with relatively preserved beta-cell function [5–8]. The association between high C-peptide levels and CVD risk was attenuated after adjustment for cardiometabolic risk factors that are typical for insulin resistance. Thus, it is plausible that high C-peptide levels reflect compensatory response to insulin resistance in the setting of relatively preserved beta-cell function, while the CVD risk is primarily driven by vascular and metabolic consequences of insulin resistance.

In contrast, those with low C-peptide levels were less obese, had relatively normal lipid levels, and required more frequent use of insulin; thus, this group appeared to share more type 1 diabetes characteristics. Consistent with our finding of higher CVD risk in our low C-peptide group, individuals with type 1 diabetes with greater beta-cell damage (as indicated by lower C-peptide levels) are at higher risk of diabetes complications [31, 32]. Similar to type 1 diabetes, reduced beta-cell function in our low C-peptide group manifested in higher glucose variation and rates of severe hypoglycemia.

Our results suggest that this phenotype (with some type 1 diabetes features) may be present in a surprising portion of patients with advanced type 2 diabetes. Consistent with the relatively more type 1 diabetes phenotype, the fasting C-peptide levels in the lower range indicated a beta-cell dysfunction not too dissimilar from type 1 diabetes (typically < 0.25 nmol/l) [33]. Some of these individuals diagnosed with type 2 diabetes may deserve to be classified within other diabetes subtypes, including hybrid forms of diabetes; slowly evolving, immune-mediated diabetes of adults, previously known as latent autoimmune diabetes of adults (LADA); ketone-prone diabetes (KPD) or monogenic defects of β-cell function [34]. In a nested case–control cohort from the ACCORD trial, those with low C-peptide levels had higher rates of testing positive for several islet antibodies and increased risk of severe hypoglycemia [14]. These findings of a spectrum of phenotypes within the broader category of type 2 diabetes is consistent with growing genetic evidence of sub-phenotypes of diabetes [35]. Recognition of these differences may affect medication selection and other health care decisions. For example, use of insulin sensitizers in this low C-peptide group may be less useful than insulin or compounds promoting insulin secretion. Although insulin supplementation may be appropriate in these individuals to improve glycemic control, it will also increase the risk of hypoglycemia and, as discussed above, may not limit glucose variation. This may therefore also be a group that may benefit more from use of continuous glucose monitoring to better adjust treatment regimens to reduce hypoglycemia and glucose fluctuations. Finally, adding medications with known cardioprotective action may help reduce the risk of CVD in this relatively higher risk group.

Our analysis also showed a significantly higher proportion of self-reported African Americans among those with low C-peptide levels. This is consistent with data from the NHANES III cohort in African Americans without diabetes [36]. In previous studies, African Americans without diabetes and black African men with early diabetes exhibited lower insulin secretion compared with their White counterparts for their given insulin sensitivity for glucose metabolism [37, 38].

This study has several strengths. Standardized protocols were used in the VADT for treatment of diabetes and other risk factors, reducing the likelihood for bias in treatment among the different quartiles of individuals. The sample size and duration of follow-up go beyond other studies for assessment of C-peptide levels, measures of glucose control and CVD assessment and adjudication. Key study metrics were measured prospectively every 3 months for the entire study duration with standardized protocols for measurement of fasting glucose, HbA_1c_ and other risk factors. The VADT also included extensive collection of data on diabetes and non-diabetes medication use, as well as careful adjudication of events, including the composite CVD outcome and severe hypoglycemia. The large sample size and number of events allowed robust statistical analyses with adjustment for many relevant covariates. The frequency of visits allowed use of time-varying estimates for glucose variation or severe hypoglycemia in the Cox models up until the CVD event.

There are several limitations in our study. The typical participant in the VADT was male with known CVD or at high risk for subsequent CVD. Therefore, it is unclear if our findings will be generalizable in cohorts with a larger proportion of females or less underlying CVD risk. Although the association between C-peptide levels and CVD was independent of baseline insulin or sulphonylureas use, we cannot dismiss the possibility that C-peptide levels may reflect more subtle variations in treatment regimen, individual anti-diabetes medications, and/or their dosage. Our study has relied on fasting measures of glycemic control at study visits and therefore cannot provide additional critical information on short-term glucose variation that will better capture and reflect postprandial changes in glucose. The VADT trial also did not collect information on participants’ compliance with behavioral measures that could have influenced diabetes outcomes. Nonetheless, C-peptide appeared a surprisingly robust/persistent independent indicator of CVD risk even among this relatively homogenous cohort of advanced type 2 diabetes patients.

In conclusion, both low and high C-peptide levels were associated with CVD risk in this population. Contrary to our hypothesis, greater glucose variation or severe hypoglycemia did not explain the association between low C-peptide levels and CVD, suggesting additional mechanisms may link low C-peptide with increased CVD. Thus, C-peptide measurement may be a useful indicator of future glycemic control patterns and CVD risk and may identify phenotypic differences that could be relevant for clinical decision making in advanced type 2 diabetes.

## Supplementary Information

**Additional file 1: Fig. S1.** Restricted cubic splines curve and 95% CI of relationship between C-peptide levels and CVD risk adjusted for glucose lowering group in all participants with baseline C-peptide levels (n=1693).

## Data Availability

The datasets used and/or analyzed during the current study are available from the corresponding author on reasonable request.
