# Supplementary material for: Association of low fasting C-peptide levels with cardiovascular risk, visit-to-visit glucose variation and severe hypoglycemia in the Veterans Affairs Diabetes Trial (VADT)
Source: Cardiovasc Diabetol. 2021 Dec 8;20:232. doi: 10.1186/s12933-021-01418-z (PMC8656002; doi:10.1186/s12933-021-01418-z)
Supplement: Supplementary file 1 — Additional file 1: Fig. S1. Restricted cubic splines curve and 95% CI of relationship between C-peptide levels and CVD risk adjusted for glucose lowering group in all participants with baseline C-peptide levels (n=1693). [file 12933_2021_1418_MOESM1_ESM.pdf]

Figure: Restricted cubic splines curve and 95% CI of relationship between C-peptide levels and CVD risk adjusted for glucose lowering group in all participants with baseline C-peptide levels (n=1,693).

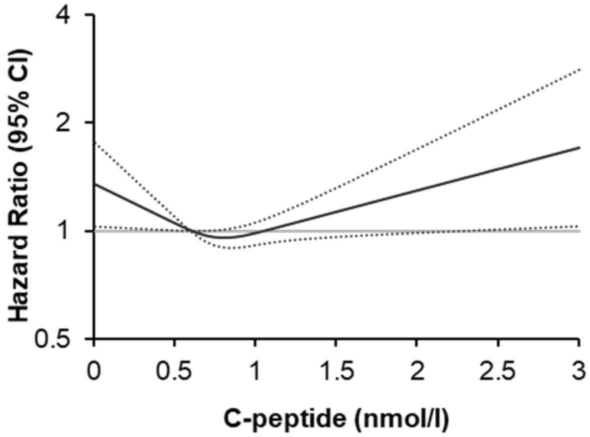

1
